# Reaction Behavior and Kinetic Model of Hydroisomerization and Hydroaromatization of Fluid Catalytic Cracking Gasoline

**DOI:** 10.3390/molecules30040783

**Published:** 2025-02-08

**Authors:** Haijun Zhong, Xiwen Song, Shuai He, Xuerui Zhang, Qingxun Li, Haicheng Xiao, Xiaowei Hu, Yue Wang, Boyan Chen, Wangliang Li

**Affiliations:** 1 Petrochemical Research Institute, PetroChina Company Limited, Beijing 102206, China; zhonghaijun@petrochina.com.cn (H.Z.); songxiwen010@petrochina.com.cn (X.S.); heshuai010@petrochina.com.cn (S.H.); zhangxuerui010@petrochina.com.cn (X.Z.); liqingxun@petrochina.com.cn (Q.L.); xhc459@petrochina.com.cn (H.X.); huxiaowei010@petrochina.com.cn (X.H.); wangyue010@petrochina.com.cn (Y.W.); chenboyan@petrochina.com.cn (B.C.); 2 CAS Key Laboratory of Green Process and Engineering, Institute of Process Engineering, Chinese Academy of Sciences, Beijing 100190, China; 3 University of Chinese Academy of Sciences, Beijing 100049, China

**Keywords:** FCC gasoline, hydro-upgrading, hydroisomerization, hydroaromatization, kinetic model

## Abstract

The hydro-upgrading reaction behavior of model compound 1-hexene and FCC middle gasoline was investigated using a fixed-bed hydrogenation microreactor with a prepared La-Ni-Zn/H-ZSM-5 catalyst. The catalyst was prepared by wetness impregnation method, using hydrothermal treated H-ZSM-5 zeolite blended with alumina as the support, and La, Ni, Zn as the active metals. The reaction tests were carried out at 300–380 °C, 1.0 MPa, 1.5–3.0 h^−1^ (LSHV), and 300:1 *v*/*v* (H_2_/oil). Analyzing the changes in hydrocarbon components before and after hydro-upgrading elucidated the mechanistic pathways of olefin hydroisomerization and hydroaromatization. Based on these findings, a seven-lump kinetic model was established for the FCC middle gasoline hydro-upgrading process. Given the diversity and complexity of reaction products, they were grouped into seven lumps: normal paraffins, isoparaffins, linear olefins, branched olefins, cycloolefins, naphthenes, and aromatics. Kinetic parameters were estimated using the Levenberg–Marquardt algorithm and validated against experimental data. The results showed that the conversion of naphthenes to aromatics exhibited the highest activation energy and pre-exponential factor, resulting in the largest reaction rate increase within the 320–380 °C range. The model accurately predicted the product yields of FCC gasoline hydro-upgrading, with a relative error of less than 5%. These findings provide valuable guidance for the optimization, design, and operation of FCC gasoline hydro-upgrading units, as well as for catalyst development, with the aim of improving process efficiency and fuel quality.

## 1. Introduction

Fluid catalytic cracking (FCC) gasoline is a primary component of the gasoline pool, typically contributing 1/3 to 4/5 of the blend used in automotive fuels. Upgrading the quality of automotive gasoline involves reducing the sulfur and olefin content of FCC gasoline while preserving its octane value. At present, there are two main types of FCC gasoline hydro-upgrading process technologies that have been industrially applied, including selective hydrogenation and desulfurization technology (such as Prime-G^+^, SCANfining, RSDS, PHG, etc.), and selective hydrogenation desulfurization and hydro-upgrading combined technology (such as OCTGAIN, ISAL, GARDES, M-PHG, etc.). Among these, GARDES and M-PHG technologies have been implemented in nearly 15 industrial units, playing a vital role in meeting National V and VI (A) gasoline quality standards [1,2,3,4,5,6]. However, with the enforcement of the National VI (B) standard for automotive gasoline, which mandates reducing olefin content to 15 vol%, there is an urgent need to develop new processes and catalyst formulations. These innovations must focus on achieving significant olefin reduction, maintaining octane value, and delivering both technical and economic advantages [7,8,9,10].

The octane number of gasoline is closely linked to its chemical composition, particularly the molecular structure of hydrocarbons. The approximate sequence of research octane number (RON) for hydrocarbons with the same carbon number is as follows: aromatics > isoparaffins > naphthenes≈olefins > *n*-paraffins. Research indicates that converting linear olefins to *n*-paraffins significantly reduces RON, whereas converting olefins to isoparaffins slightly increases RON, depending on the degree of isomerization. The more branched and compact the molecular structure, the higher the RON. Therefore, converting olefins in FCC gasoline into aromatics and highly branched isoparaffins is beneficial for reducing olefins while maintaining RON [11]. Developing efficient catalysts and optimizing reaction conditions are crucial for the development of FCC gasoline hydro-upgrading technology. Olefin hydroisomerization and hydroaromatization catalysts are primarily metal–acid bifunctional catalysts. Metal components, such as Mo, Ni, and Co oxides, provide hydrogenation and dehydrogenation centers, while acidic carriers, such as ZSM-5, β, L, MCM-22, and SAPO-11 molecular sieves, supply acid sites [12,13,14,15,16,17]. A recent research hotspot in FCC gasoline hydro-upgrading is the development of catalysts that enhance olefin reduction, minimize the formation of light components and heavy aromatics, improve gasoline yield, and extend catalyst service life. Several recent studies have introduced innovative approaches and valuable theoretical insights. For instance, Pan et al. [18] prepared Zn-modified ZSM-5 zeolite-supported Ni bifunctional catalysts for FCC gasoline hydro-upgrading. Among them, Zn-Ni-ZSM-5 with the closest intimacy between ZnOH^+^ and Ni^0^ exhibited the highest iso-alkane selectivity, while Ni/Zn-ZSM-5 with the least intimacy showed the highest aromatics selectivity. Huang et al. [19] successfully developed a Ni_2_P/SAPO-11 bifunctional catalyst using incipient impregnation followed by temperature-programmed hydrogen reduction. This catalyst achieved complete conversion of thiophene and 1-hexene with 74% selectivity toward branched isomers under conditions of 320 °C, 1.5 MPa, and an H_2_/oil ratio of 750 (*v*/*v*). Additionally, Wang et al. [20] utilized density functional theory (DFT) to study the active centers for C_6_ olefin aromatization on a Zn^2+^/H-ZSM-5 catalyst, revealing that the Lewis acid sites serve as dehydrogenation centers, while Brönsted acid sites act as cyclization centers. Wei et al. [21] further explored the effect of mesopore spatial distribution in H-ZSM-5 catalysts on zinc states and product distribution during 1-hexene aromatization, offering insights into how mesopores in Zn/H-ZSM-5 influence xylene selectivity. These findings collectively contribute to advancing FCC gasoline hydro-upgrading catalyst development and optimizing catalytic performance.

FCC gasoline hydroisomerization and hydroaromatization involve complex reaction systems designed to achieve multi-objective catalysis. For instance, the olefin hydroaromatization reaction encompasses cracking, oligomerization, cyclization, hydrogen transfer, and dehydrogenation, resulting in the co-production of aromatics and by-products. In-depth research into the reaction mechanisms of FCC gasoline hydro-upgrading processes, along with the development of kinetic models, is crucial for guiding process design, optimizing equipment operations, and advancing catalyst development. Some studies have provided valuable insights into this area. For example, Fan et al. [22] proposed a reaction mechanism for olefin hydroisomerization and aromatization using a Ni-Mo/H-ZSM-5+Al_2_O_3_ catalyst. According to their findings, (1) olefins adsorbed on acid sites are converted into carbocations, which are subsequently transformed into *i*-paraffins through rearrangement, isomerization, and hydrogen spillover; and (2) olefins are converted into dienes, cycloolefins, and aromatics via hydrogen transfer, cyclization, and dehydrogenation. They also developed a six-lump kinetic model to predict the product yields of FCC gasoline hydro-upgrading, which showed good agreement with experimental results [23].

Although some lump kinetic models reported in the literature have well reflected the laws of FCC gasoline hydroisomerization or hydroaromatization reactions, and can effectively predict the hydrocarbon composition of the product. However, they often fail to consider the complete reaction network, limiting their utility for simulating and optimizing the entire FCC gasoline hydro-upgrading process. For example, some studies [23,24,25] did not account for the conversion of *n*-paraffins and naphthenes into isoparaffins or the interconversion between naphthenes and aromatics. Some studies [26,27] considered these reactions, but they treated olefins as a single group rather than subdividing them into linear olefins (L-O), branched olefins (B-O), and cycloolefins (C-O), thus failing to provide detailed conversion information for different olefin types.

This study investigates the hydro-upgrading reaction process of the olefin model compound 1-hexene and real FCC gasoline middle fractions. By examining the hydrogenation, hydroisomerization, and hydroaromatization behaviors of olefins, a hydrocarbon conversion reaction network is constructed. Based on this, a lumped kinetic model for FCC gasoline hydro-upgrading is established using the lumped kinetic method, and the reaction rate constants and activation energies for each step in the reaction network are calculated. Finally, the reliability of the model calculations is thoroughly analyzed.

## 2. Results and Discussion

### 2.1. Reaction Behavior of Hydroisomerization and Aromatization of FCC Gasoline

#### 2.1.1. Reaction Behavior of Olefin Model Compound

To gain mechanistic insights into the isomerization and aromatization reaction pathways of olefins in FCC gasoline on hydro-upgrading catalysts, the conversion behavior of a representative olefin model compound, 1-hexene, was investigated. The results of these studies are summarized in Table 1, Table 2 and Table 3.

Under the four reaction conditions listed in Table 1, the conversion rate of 1-hexene all reaches 100%, yielding a variety of products, including *n*-paraffins, *i*-paraffins, linear olefins, branched olefins, cycloolefins, naphthenes, and aromatics. At 300 °C + 1.5 h^−1^, the total content of branched olefins is 3.7 times that of linear olefins, and C_6_ branched olefins account for up to 38.44 wt%, indicating 1-hexene underwent significant skeletal isomerization reaction, which only needs weak or moderate acid centers; the used La-Ni-Zn/H-ZSM-5 catalyst in this article can provide suitable molecular sieve acidic centers. The products primarily consist of C_3_–C_6_ and C_6_^+^ compounds. The formation of C_4_, C_5_, and C_6_^+^ compounds suggests that dimerization and subsequent cracking reactions occur during the process [28]. This reaction’s progress aligns with the bimolecular cracking mechanism: Two hexene molecules first oligomerize on the acidic sites within the H-ZSM-5 channels to form C_12_ carbocations, converted into tertiary carbocations by skeletal isomerization. Tertiary carbocations are highly prone to cracking reactions after formation, leading to their conversion into two smaller olefin molecules, including C_3_^=^+C_9_^=^, C_4_^=^+C_8_^=^, and C_5_^=^+C_7_^=^. Additionally, C_7_-C_9_ olefins are also prone to secondary cracking reactions, leading to the conversion into propylene, *n*-butene, isobutene, *n*-pentene, isopentene, etc. [29,30,31,32]. The intensity of the secondary cracking reactions increases with rising temperatures. As shown in Table 2, nearly no C_7_ or higher olefins are present in the product at 380 °C. During this process, some C_3_–C_9_ olefins are converted into corresponding *n*-paraffins and *i*-paraffins through hydrogenation saturation, highlighting the complex interplay of olefins oligomerization, cracking, isomerization, and hydrogenation in the FCC gasoline hydro-upgrading.

In addition, the main conversion products of 1-hexene at 300 °C + 1.5 h^−1^ are branched olefins and linear olefins, along with a small amount of cycloolefins and naphthenes. However, no aromatics were detected, indicating that olefin cyclization is easier than aromatization. The content of light hydrocarbons (≤C_3_) and aromatics in the product increases synchronously with rising reaction temperatures, reaching the highest values of 14.18 wt% and 25.65 wt% at 380 °C, respectively. Table 2 shows nearly all of the light hydrocarbons are saturated paraffins, differing from the Ni-Mo/H-ZSM-5+Al_2_O_3_ catalyst system, which reported 90% of the light hydrocarbons were C_3_ olefins in the 1-hexene hydro-upgrading product [22], indicating that the La-Ni-Zn/H-ZSM-5 catalyst prepared in this study exhibits strong C_2_–C_3_ olefin hydrogenation saturation activity. Table 2 also shows the aromatics in the product dominated by alkylbenzenes with only a little benzene at 380 °C, suggesting that benzene acts as an intermediate, undergoing alkylation reactions [33].

Comparing the product distribution at LSHV of 1.5 h^−1^ and 3.0 h^−1^ at the same reaction temperature of 340 °C (Table 3) indicates that an increase in the LSHV reduced the residence time of 1-hexene and intermediates on catalytic active sites, which results in decreased catalyst activity of cracking, isomerization, and aromatization. Consequently, the content of branched olefins, cycloolefins, and naphthenes as intermediates increases significantly, while the content of light hydrocarbons, isoparaffins, and aromatics decreases markedly. These findings support this olefin hydroaromatization mechanism: 1-hexene molecules undergo hydrogen transfer at the acid center to form dienes, which are then sequentially converted to cycloolefins, naphthenes, and aromatics through hydrogen transfer, cyclization, and dehydrogenation processes [22].

According to the research results on the conversion behavior of 1-hexene, overall, the olefin skeletal isomerization is dominant at low reaction temperatures, while the hydrogenation saturation is elevated with the increasing reaction temperature, and the aromatization activity is promoted at higher temperature.

#### 2.1.2. Reaction Behavior of FCC Middle Gasoline

The carbon number distribution and group composition of the FCC middle gasoline are presented in Table 4. The hydrocarbon group composition and RON changes in hydro-upgrading products at different reaction temperatures are presented in Table 5. The data reveal that at varying reaction temperatures, the content of P (*n*-paraffins), I (*i*-paraffins), N (naphthenes), and A (aromatics) in the products was higher than in the feedstock, while the content of L-O (linear olefins), B-O (branched olefins), and C-O (cycloolefins) was lower. This indicates that the hydroisomerization and aromatization processes in FCC gasoline primarily involve the conversion of olefins.

**Figure 1:**
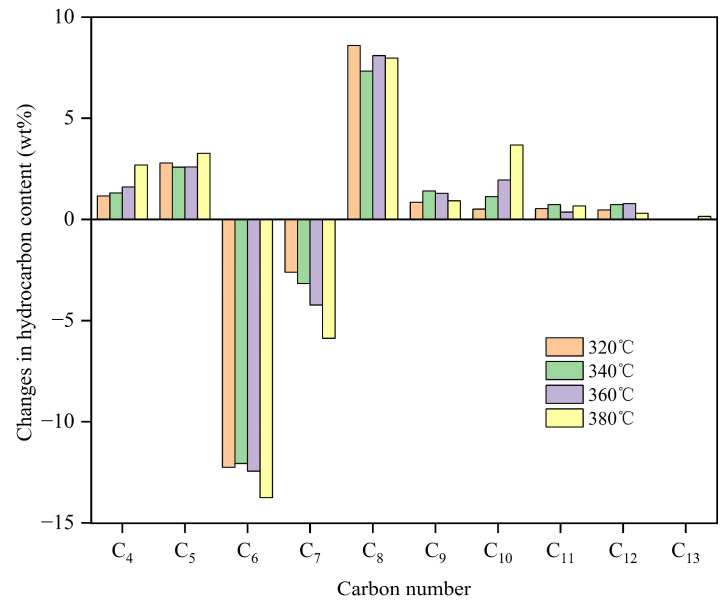
Changes in hydrocarbon content with different carbon numbers of hydro-upgrading products.

Figure 1 illustrates the changes in the content of hydrocarbons with different carbon numbers in the hydro-upgrading products at various reaction temperatures. As shown in Figure 1, the content of C_4_ and C_5_ hydrocarbons increases after the hydro-upgrading reaction under different conditions, and this increase is proportional to the reaction temperature. This is attributed to the cracking of C_6_–C_7_ olefins, which are present in the highest concentrations in FCC middle gasoline. The higher the temperature, the more extensive the cracking reactions become. Additionally, the content of C_8_ and higher hydrocarbons increases across all conditions, with the most notable rise observed in C_8_. This is attributed to C_6_–C_7_ olefins undergoing a combination of cracking, polymerization, hydrogen transfer, cyclization, dehydrogenation, and alkylation processes.

To clarify the reaction patterns, further analysis was conducted on the changes in hydrocarbon group composition with different carbon numbers. Table 6 summarizes the variations in P (*n*-paraffins), I (*i*-paraffins), N (naphthenes), and A (aromatics). Table 7 shows the detailed changes in L-O (linear olefins), B-O (branched olefins), and C-O (cycloolefins). According to the data in Table 6, both *n*-paraffins and *i*-paraffins showed increases at different reaction temperatures, with gains of 2.48–3.10 wt% and 1.96–5.64 wt%, respectively. The increase initially rose and then declined, peaking at 360 °C. As the reaction temperature increased, C_4_ and C_5_ *n*-paraffins and *i*-paraffins increased progressively, while C_8_ *n*-paraffins gradually decreased. C_6_–C_8_ *i*-paraffins first rose and then fell, peaking at 360 °C. In the 320–360 °C range, observed minimal changes for C_6_ and C_7_ *n*-paraffins (<0.5 wt%), as well as C_9_ and higher *n*-paraffins/*i*-paraffins.

For naphthenes and aromatics, increases of 0.73–2.15 wt% and 2.36–11.40 wt%, respectively, were observed. The increase in naphthenes peaked at 340 °C before declining, with C_6_, C_7_, and C_9_ naphthenes showing similar gains at 340 °C and 360 °C. However, C_8_ naphthenes began to decrease at 360 °C, and at 380 °C, C_6_ and C_7_ naphthenes exhibited minimal or no growth. Aromatic composition changes showed that C_6_ aromatics consistently decreased by ~1.0 wt%, while C_7_ aromatics showed negligible increases. Conversely, C_8_ and C_9_ aromatics increased with rising temperatures, with C_8_ aromatics exhibiting a more pronounced growth than C_9_ aromatics. These trends suggest that at 360–380 °C, dehydrogenation and the conversion of naphthenes to aromatics are accelerated, with C_6_ and C_7_ naphthenes requiring 380 °C for conversion, while C_8_ naphthenes conversion can take place at 360 °C.

The total olefin content decreased at all reaction temperatures, with the degree of reduction proportional to both the reaction temperature and the initial olefin content. The reduction followed the trend: branched olefins (5.20–10.80 wt%) > linear olefins (1.83–5.93 wt%) > cycloolefins (1.84–2.32 wt%). C_4_ olefins increased in all products, remaining as linear olefins with gains of 0.41–0.88 wt%, which were inversely proportional to the reaction temperature. In the 320–360 °C range, C_5_ linear and branched olefins increased by 0.11–0.42 wt% and 0.57–1.73 wt%, respectively, but at 380 °C, changes in C_5_ olefins were negligible.

Among the olefins in FCC middle gasoline, C_6_ and C_7_ had the highest initial content, and their changes during hydro-upgrading were the most significant. Table 8 presents the content of various C_6_ and C_7_ olefin structures in the hydro-upgrading products at different temperatures. C_6_ and C_7_ linear olefins, branched olefins, and cycloolefins all decreased with increasing temperatures. The content of internal olefins was higher than terminal olefins, but terminal olefins exhibited greater reactivity than internal olefins. Similarly, linear olefins were more reactive than branched olefins. Additionally, for olefins with the same structure, C_6_ olefins were more reactive than C_7_ olefins.

### 2.2. Lumped Kinetic Model for FCC Middle Gasoline Hydro-Upgrading

#### 2.2.1. Establishment of a Lumped Reaction Kinetics Model

**Figure 2:**
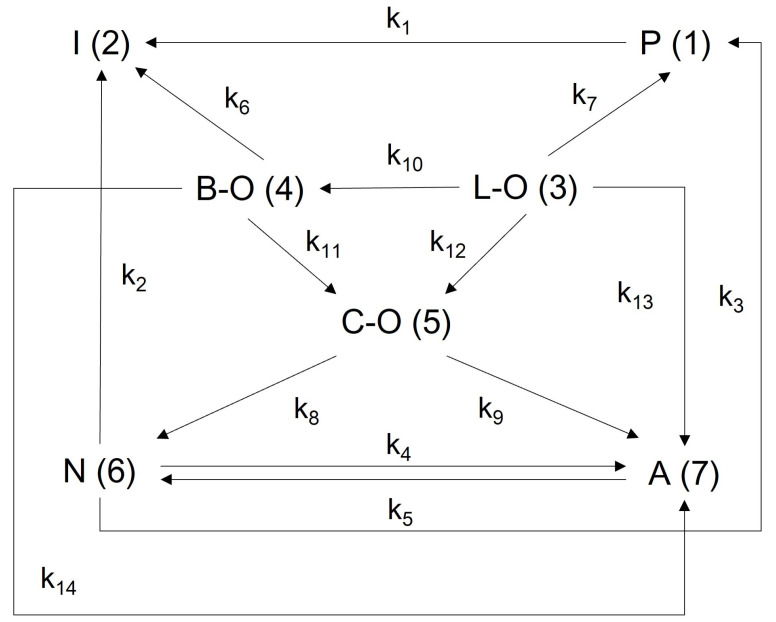
Lumped reaction network for FCC middle gasoline hydro-upgrading.

To comprehensively and accurately characterize the kinetic properties of the FCC gasoline hydro-upgrading reaction network, FCC middle gasoline was selected as the research object. The hydrocarbon species in FCC middle gasoline were categorized into seven lumps: *n*-paraffins (P), *i*-paraffins (I), naphthenes (N), aromatics (A), linear olefins (L-O), branched olefins (B-O), and cycloolefins (C-O). Based on these categories, a lumped reaction network for FCC middle gasoline hydro-upgrading was developed, as illustrated in Figure 2.

In the reaction network, the conversion pathways for each lumped hydrocarbon group are as follows: L-O can be converted to P, B-O, C-O, and A through hydrogenation, isomerization, and cyclization-dehydrogenation, respectively. B-O can be converted to I, C-O, and A via hydrogenation and cyclization-dehydrogenation. C-O can be converted to N and A through hydrogenation and dehydrogenation, respectively. P can be converted to I through isomerization. N can be converted to P and I by cracking and can also interconvert with A through dehydrogenation and hydrogenation.

Given that the liquid yield of FCC gasoline hydro-upgrading exceeds 98 wt% [4,27], the gasses produced by gasoline cracking were ignored in this study. To simplify the kinetic model, the following assumptions were made: (1) All reactions are first-order reactions. (2) The reactor behaves as a plug flow reactor. (3) The published experimental results [9] and industrial application [3,4] have shown that the catalyst used in this study has good activity stability, and therefore its activity decay in the FCC gasoline hydro-upgrading experiment can be ignored. (4) The reaction process is isothermal and isobaric. Based on these assumptions, the kinetic equations for the lumped reaction model were established, providing a mathematical framework to describe the hydro-upgrading process. According to the continuity equation,

(1) $${\left(\frac{\partial \rho a_{i}}{\partial t}\right)}_{x}+G_{v}{\left(\frac{\partial a_{i}}{\partial x}\right)}_{t}=r_{i}$$

where ρ is the density of the oil/gas mixture, g/cm^3^; $a_{i}$ is the concentration of the lump i, g·g^−1^(gas); t is the reaction time, h; $G_{v}$ is the surface (cross-section) flow rate of the reacting gas, g·cm^−2^·h^−1^; x is the distance from the reactor inlet into the reactor, cm; $r_{i}$ is the reaction rate of the lump i, g·cm^−3^·h^−1^.

The density of the oil/gas mixture in the reaction can be expressed by the following equation:

(2) $$\rho =\frac{P\bar{M}}{RT}$$

where P is the reaction pressure, Pa; $\bar{M}$ is the average relative molecular weight of the oil/gas mixture, g/mol; R is the universal gas constant, 8.314 J/(mol·K); T is the reaction temperature, K.

The surface (cross-section) flow rate of the reacting gas is proportional to the linear velocity of the material in the reactor:

(3) $$G_{v}=\rho u$$

where u is the linear velocity of the material, cm/h.

For each individual reaction, the rate of disappearance of lump i in the first-order reaction is proportional to the molar concentration of lump I ($\rho a_{i}$) and the density of the catalyst to the volume of gas ($\frac{\rho_{c}}{\varepsilon }$), then the reaction rate for the lump i is as follows:

(4) $$r_{i}=-k_{i}(\rho a_{i})(\frac{\rho_{c}}{\varepsilon })$$

where $k_{i}$ is the reaction rate constant for the lump i, cm^3^·g^−1^·s^−1^; ε is the bed porosity.

The airspeed of the reacting gas is defined as Equation (5):

(5) $$S_{wh}=(\frac{G_{v}\varepsilon }{\rho_{c}H})$$

where H is the total height of the catalyst bed, cm.

The dimensionless height of a certain cross-section at reactor inlet distance x can be expressed as follows:

(6) $$X=\frac{x}{H}$$

Then, the expression of the differential equation for the kinetic model of lump i can be derived as shown in Equation (7).

(7) $$\frac{da_{i}}{dX}=-\frac{1}{S_{WH}}\rho k_{i}a_{i}=-\frac{P\bar{M}}{S_{WH}RT}k_{i}a_{i}$$

Based on the established reaction network of FCC middle gasoline hydro-upgrading reaction (Figure 1), the expression differential equations of the kinetic model for each lump can be derived as shown from Equation (8) to Equation (14).

(8) $$\frac{da_{1}}{dX}=-\frac{P\bar{M}}{S_{WH}RT}\left(k_{1}a_{1}{-k}_{7}a_{3}-k_{3}a_{6}\right)$$

(9) $$\frac{da_{2}}{dX}=-\frac{P\bar{M}}{S_{WH}RT}\left(-k_{1}a_{1}-k_{6}a_{4}-k_{2}a_{6}\right)$$

(10) $$\frac{da_{3}}{dX}=-\frac{P\bar{M}}{S_{WH}RT}\left(k_{7}a_{3}+k_{10}a_{3}+k_{12}a_{3}+k_{13}a_{3}\right)$$

(11) $$\frac{da_{4}}{dX}=-\frac{P\bar{M}}{S_{WH}RT}\left(k_{6}a_{4}+k_{11}a_{4}+k_{14}a_{4}-k_{10}a_{3}\right)$$

(12) $$\frac{da_{5}}{dX}=-\frac{P\bar{M}}{S_{WH}RT}\left(k_{8}a_{5}+k_{9}a_{5}-k_{11}a_{4}-k_{12}a_{3}\right)$$

(13) $$\frac{da_{6}}{dX}=-\frac{P\bar{M}}{S_{WH}RT}\left(k_{2}a_{6}+k_{3}a_{6}+k_{4}a_{6}-k_{8}a_{5}-k_{5}a_{7}\right)$$

(14) $$\frac{da_{7}}{dX}=-\frac{P\bar{M}}{S_{WH}RT}\left(k_{5}a_{7}-k_{13}a_{3}-k_{14}a_{4}-k_{9}a_{5}-k_{4}a_{6}\right)$$

where $a_{1}$, $a_{2}$, $a_{3}$, $a_{4}$, $a_{5}$, $a_{6}$, and $a_{7}$ represent the concentrations of the lump P, I, L-O, B-O, C-O, N, and A, respectively.

#### 2.2.2. Calculation and Analysis of Kinetic Parameters

In this work, MATLAB R2021b software was used to calculate the parameters of the lumping kinetic model. Due to the high accuracy of the Runge–Kutta algorithm, this method is used to solve the differential kinetic equations, and the built-in function ode45 was chosen as the integration function. The Levenberg–Marquardt algorithm based on the least squares method has fast convergence speed, good stability, high solution accuracy, strong universality, and is computationally resource friendly. Therefore, this method is adopted to optimize the objective function, and the solution function was chosen as lsqnonlin. The optimal solution of kinetic equations was obtained by iterative computations of the reaction rate constant k. The objective function is as follows:

(15) $$Function=\sum_{i=1}^{n}{{(y}_{ie}-y_{ic})}^{2}$$

where $y_{ie}$ represents the experimental value for the lump i of reactor outlet products, $y_{ic}$ represents the calculated value of the lump i of reactor outlet products.

After obtaining the rate constant k for each reaction at different temperatures, the activation energy and the pre-exponential factor for each reaction were calculated by fitting the Arrhenius equation as shown in Equation (16).

(16) $$k=Aexp(-\frac{Ea}{RT})$$

where A is the pre-exponential factor, cm^3^·g^−1^·h^−1^; Ea is the apparent activation energy of the reaction, kJ·mol^−1^.

Based on the experimental data of the FCC middle gasoline hydro-upgrading reaction at 320–380 °C shown in Table 4, Table 5, Table 6, Table 7 and Table 8 and the above calculations, the kinetic parameters such as the rate constant k, the activation energy Ea, and the pre-exponential factor A of each reaction in the lumping kinetic network were obtained, and the results of calculations were shown in the following Table 9.

The analysis of kinetic model parameters in Table 9 reveals the following insights:

Temperature Sensitivity of Rate Constants: All reaction rate constants increase with rising reaction temperature. Among these, the rate constants k_4_, k_5_, k_6_, k_7_, k_8_, k_9_, k_13_, and k_14_—which correspond to the generation of aromatics (A), the conversion of B-O to I, L-O to P, L-O to B-O, and C-O to N—are particularly sensitive to temperature changes. In contrast, the rate constants k_3_, k_11_, and k_12_, which govern the conversion of N to P, B-O to C-O, and L-O to C-O, are less temperature-sensitive.

Conversion of B-O and L-O to C-O: The reaction rate constants k_11_ and k_12_ for the conversion of B-O and L-O to C-O remain close to zero at all temperatures, showing minimal variations. Meanwhile, k_10_, representing the conversion of L-O to B-O, is relatively low at 320 °C but increases sharply with rising temperature. This suggests that the conversion of L-O to B-O is the primary pathway for internal olefin transformation.

Sources of N (Naphthenes): The main sources of N are C-O and A. Among these, the reaction rate constant k_8_ (C-O to N) is the highest across all temperatures, followed by k_9_ (C-O to A). However, due to the low initial content of C-O (2.39 wt%) in the feedstock, the overall increase in N is limited. Additionally, the conversion of A to N is the reverse of the N to A reaction. At 320 °C, the rate constant k_5_ (A to N) is significantly higher than k_4_ (N to A), but as the temperature rises beyond 340 °C, k_4_ and k_5_ approach similar values.

Temperature-Dependent Behavior of N: The rate constants k_2_ and k_3_, representing the conversion of N to I and P, are relatively large. This results in N content at 340 °C being similar to that at 320 °C, with the rate of N content increase slowing after 340 °C. These observations highlight the competitive and interdependent nature of the reactions involving N at different temperatures.

The main sources of *n*-paraffins (P) are linear olefins (L-O) and naphthenes (N). Since the reaction rate constant k_3_ for the conversion of N to P and the content of N at each temperature are relatively small, the contribution of the N to P reaction to the increase in P content is limited. On the other hand, the conversion of L-O to P competes with the conversion of L-O to aromatics (A). At 320 °C, the rate constant k_7_ for the L-O to P reaction is significantly larger than that for the L-O to A reaction (k_13_), resulting in a noticeable increase in P content. The reaction rate constant k1 for the conversion of P to isoparaffins (I) is relatively small, further contributing to the accumulation of P at lower temperatures. However, as the temperature increases, the difference between k_13_(L-O to A) and k_7_ (L-O to P) gradually diminishes, with k_13_ surpassing k_7_ after 360 °C. At this stage, L-O is more likely to convert to A, and the reaction rate constant k_1_ for P to I also increases. This causes the rate of increase in P content to slow down between 320 °C and 360 °C and eventually decline after 360 °C.

The main sources of isoparaffins (I) are branched olefins (B-O), *n*-paraffins (P), and naphthenes (N). The rate constants k_1_ and k_2_, which govern the conversion of P to I and N to I, respectively, are small across all temperatures. Consequently, the conversion of B-O to I remains the primary pathway for I production. However, this reaction competes with the conversion of B-O to aromatics (A), which is governed by k_14_. The reaction rate constant k_14_ for B-O to A is larger than k6 for B-O to I at all temperatures. However, the difference between k_6_ and k_14_ initially increases with rising temperature and then decreases, reaching its smallest value at 340 °C. At this temperature, part of A is also converted to N, further influencing the reaction network. As a result, the increase in I content is slightly greater than that of A at 340 °C. At other temperatures, the increase in I content is smaller than that of A, and the rate of I content increase follows a trend of first rising and then declining as the temperature increases.

The main sources of aromatics (A) are cycloolefins (C-O), branched olefins (B-O), linear olefins (L-O), and naphthenes (N). Among these, the reaction rate constant k_9_ for the conversion of C-O to A is the largest across all temperatures. The rate constants k_4_, k_9_, k_13_, and k_14_, corresponding to the conversion of N, C-O, L-O, and B-O to A, respectively, show significant increases with rising temperature.

The rate of increase in k_14_ (B-O to A) relative to the competing k_6_ (B-O to I) decreases within the 320–340 °C range but increases with temperature beyond this range. Similarly, the rate constants for reactions generating A relative to their competing reactions generally increase with temperature. Additionally, the difference between k_4_ (N to A) and its inverse reaction gradually narrows as temperature increases, indicating that higher temperatures favor the generation of A. Consequently, the content of A shows a consistent upward trend at all temperatures.

The reaction activation energy represents the energy barrier, while the pre-exponential factor reflects the availability of active sites on the catalyst surface [34]. According to the activation energy and pre-exponential factor values in Table 9, the reaction converting N to A exhibits the highest activation energy, indicating that the aromatization reaction is strongly influenced by temperature and faces the highest energy barrier. This is because aromatization is an endothermic reaction, and increasing the temperature promotes its progress. Additionally, this reaction has the highest pre-exponential factor, suggesting an abundance of active sites on the catalyst surface for aromatization. Consequently, k_4_ is highly temperature-sensitive, with the largest rate of increase observed within the 320–380 °C range. Conversely, the reaction converting N to P has the lowest activation energy, as it is an exothermic reaction. However, the corresponding pre-exponential factor is also the lowest, indicating a scarcity of active sites on the catalyst surface, which makes k_3_ less sensitive to temperature variations. These findings highlight the contrasting catalytic dynamics that govern the conversion of N to A and N to P, emphasizing the distinct influences of reaction energetics and catalyst surface activity.

### 2.3. Validation of the Kinetics Model

The calculated kinetic parameters were incorporated into the differential kinetic equations, and the reactor outlet compositions at each temperature were determined through numerical integration. The relative errors (REs) between the calculated and experimental values were evaluated, with the results presented in Table 10.

As shown in the table, all absolute RE values are less than 5%, indicating that the kinetic parameter calculations are both accurate and reliable. These results demonstrate that the developed kinetic model effectively predicts the reaction behavior, providing valuable guidance for optimizing the operation of FCC gasoline hydro-upgrading units.

## 3. Experimental

### 3.1. Materials and Chemicals

Lanthanum nitrate (La(NO_3_)_3_·6H_2_O), Nickel nitrate (Ni(NO_3_)_2_·6H_2_O), Zinc nitrate (Zn(NO_3_)_2_·6H_2_O), nitric acid, and 1-hexene were purchased from Sinopharm Chemical Reagent Co., Ltd. (Beijing, China). All the chemicals were of analytical grade and used without further purification. Pseudo boehmites were obtained from the Shandong Xingdu Petrochemical Technology CO., Ltd. (Zibo, China). The full-range FCC gasoline sample was sourced from the 0.9 Mt/a FCC gasoline hydrodesulfurization unit at PetroChina Harbin Petrochemical Company (Harbin, China).

### 3.2. Experimental Apparatus and Catalyst

The hydro-upgrading catalyst, La-Ni-Zn/H-ZSM-5, was prepared as follows: H-ZSM-5 (SiO_2_/Al_2_O_3_ = 30) was kneaded and extruded with pseudo boehmite as a binder and dried at 120 °C for 4 h, then calcined at 550 °C for 4 h, denoted as ZSM-5 sample. ZSM-5–550 were obtained by hydrothermal treated as-prepared ZSM-5 samples at 550 °C for 3 h at weight hourly space velocity 1.33 h^−1^ of water steam. The ZSM-5 supported metal catalysts (La-Ni-Zn/H-ZSM-5) were prepared by wetness impregnation method using the mixture solution of La_2_(NO_3_)_3_·xH_2_O,Zn(NO_3_)_2_·6H_2_O, Ni(NO_3_)_2_·6H_2_O over ZSM-5–550. The samples were dried at 120 °C for 4 h and calcined at 500 °C for 4 h. The main properties of the catalyst are summarized in Table 11. A total of 2.7 g of the catalyst was loaded into the microreactor for the experiments.

The hydro-upgrading experiments of 1-hexene or FCC middle gasoline were conducted in a fixed-bed hydrotreating microreactor with an internal diameter of 10 mm (Tianjin Golden Eagle Technology Co., Ltd. Tianjin, China). The experimental apparatus included a gas-feeding system controlled by a mass flowmeter, a syringe pump for liquid feeding, a fixed-bed reactor, and an online analysis system. The catalysts were pre-sulfided using a mixture of 3 wt% CS_2_ in cyclohexane under a pure H_2_ atmosphere. The sulfidation process was carried out at 230 °C for 8 h and then at 300 °C for 6 h, with a heating rate of 30 °C/h. After sulfidation, 1-hexene or FCC middle gasoline was fed into the reactor at predetermined flow rates once the temperature was raised to the reaction temperature. The reaction products were collected online and analyzed. For 1-hexene feed, the reaction conditions were as follows: reaction temperature of 300–380 °C, pressure of 1.0 MPa, volumetric space velocity of 1.5–3.0 h^−1^, and volumetric ratio of H_2_ to 1-hexene of 300:1. For gasoline feed, the reaction conditions were as follows: reaction temperature of 320–380 °C, pressure of 1.0 MPa, volumetric space velocity of 1.5 h^−1^, and volumetric ratio of H_2_ to gasoline of 300:1.

### 3.3. Feedstock and Product Analysis Methods

The hydrocarbon compositions of the feedstocks and products were analyzed using an Agilent 7890 Gas Chromatograph (Agilent Technologies, Santa Clara, CA, USA) equipped with a flame ionization detector (FID), a PIONA (*n*-paraffin, *i*-paraffin, olefin, naphthene, and aromatics) capillary column (50 m × 0.2 mm), and a data processing software(Ver. E.07.01, Beijing Keluo Institute of Technology, Beijing, China). The research octane number (RON) of the gasoline were calculated by the above software.

The FCC gasoline had a sulfur content of 196.0 mg/kg, a density of 717.5 kg/m^3^, and a RON value of 89.74, respectively. The carbon number distribution and group composition of the FCC gasoline are shown in Table 12. A real boiling point distiller (Dist D-2892 CC 20L, i-Fischer, Lauda-Königshofen, Germany) was used to fractionate the full-range FCC gasoline into its constituent fractions, yielding the middle gasoline fraction with a boiling point range of 55–120 °C. The mass yield of the light, middle, and heavy fractions was 27%, 37%, and 36%, respectively. The FCC middle gasoline had a sulfur content of 155.4 mg/kg, a density of 708.9 kg/m^3^, and a RON value of 84.02, respectively.

## 4. Conclusions

The hydro-upgrading of 1-hexene and FCC middle gasoline was studied using a prepared a La-Ni-Zn/H-ZSM-5 catalyst. The catalyst does not contain precious metals and the preparation method is relatively simple. For 1-hexene hydro-upgrading progress, hydroisomerization and hydaoaromatization occur through two pathways: (1) polymerization on acid centers to form C_12_ carbocations, followed by cracking, hydrogenation to produce isoparaffins; (2) hydrogen transfer to form dienes, further converted into naphthenes and aromatics via cyclization and dehydrogenation. Overall, olefin skeletal isomerization is dominant at low temperatures, the hydrogenation saturation is elevated with the increasing reaction temperature, and the aromatization activity is promoted at higher temperatures. For FCC middle gasoline hydro-upgrading progress, olefin hydrogenation, isomerization, cyclization, and aromatization significantly reduce olefin while increasing *n*-paraffins, *i*-paraffins, naphthenes, and aromatics. The rise in isoparaffins and aromatics offsets the RON loss caused by olefin reduction, maintaining gasoline quality.

A seven-lump kinetic model was developed to describe the hydro-upgrading reaction network. Seven lumps include *n*-paraffins, *i*-paraffins, naphthenes, aromatics, linear olefins, branched olefins, and cycloolefins, with 14 kinetic constants representing key reaction pathways. Kinetic parameters, including rate constants and activation energies, were estimated using the Levenberg–Marquardt algorithm. The results showed that the conversion of naphthenes to aromatics exhibited the highest activation energy and pre-exponential factor, indicating that the aromatization reaction faces the highest energy barrier as well as the existing abundance of aromatization active sites on the catalyst surface. Consequently, this reaction showed highly temperature-sensitive, resulting in the largest reaction rate increase within the 320–380 °C range. Additionally, the model accurately predicted the product yields of FCC gasoline hydro-upgrading, with a relative error of less than 5%. This comprehensive model captures the intricate reaction mechanisms and temperature dependencies of FCC gasoline hydro-upgrading. It offers a reliable tool for process optimization, catalyst development, and improving unit performance. By reducing olefin content while maintaining octane values, the model ensures efficient and high-quality gasoline production.

## Figures and Tables

**Table 1:** Reaction results of 1-hexene at different hydro-upgrading reaction conditions *.

Reaction Temperature and LHSV	Conversion (%)	≤C_3_ in Product (wt%)	Group Composition in Product (wt%)						
			P	I	L-O	B-O	C-O	N	A
300 °C + 1.5 h^−1^	100	1.89	10.28	10.73	16.42	60.77	0.07	1.73	0
340 °C + 1.5 h^−1^	100	7.66	27.05	42.99	0.66	4.63	5.80	5.28	13.59
380 °C + 1.5 h^−1^	100	14.18	29.98	39.18	0.54	0.76	0.03	3.86	25.65
340 °C + 3.0 h^−1^	100	3.95	13.91	27.07	5.19	28.13	9.31	12.00	4.39

* P—*n*-paraffins; I—*i*-paraffins; L-O—linear olefins; B-O—branched olefins; C-O—cycloolefins; N—naphthenes; A—aromatics; The other reaction conditions: P =1.0 MPa; V(H_2_)/V(1-hexene) = 300:1.

**Table 2:** Carbon number distribution in the product with 1-hexene as feedstock at 300 °C and 380 °C *.

Carbon Number	Group Composition in Product (wt%) (T = 300 °C)							Group Composition in Product (wt%) (T = 380 °C)						
	P	I	L-O	B-O	C-O	N	A	P	I	L-O	B-O	C-O	N	A
≤C_3_	1.89	0	0	0	0	0	0	14.03	0	0.15	0	0	0	0
C_4_	0.54	0.37	2.57	1.99	0	0	0	9.65	14.37	0.29	0.42	0	0	0
C_5_	0.48	0.38	1.10	4.07	0	1.47	0	4.43	10.96	0.03	0.24	0	0.06	0
C_6_	6.10	6.18	5.23	38.44	0	0	0	1.42	7.50	0	0.10	0.01	0.04	0.04
C_7_	0.33	1.87	0.52	5.63	0	0.02	0	0.23	4.67	0	0	0.02	1.88	4.99
C_8_	0	0.27	0.05	5.27	0.07	0.05	0	0.22	1.27	0	0	0	1.26	7.96
C_9_	0.18	0.47	0.57	3.14	0	0.07	0	0	0.38	0.07	0	0	0.46	5.78
≥C_10_	0.76	1.19	6.38	2.23	0	0.12	0	0	0.03	0	0	0	0.16	6.88

* The other reaction conditions: P =1.0 MPa; LHSV = 1.5 h^−1^; V(H_2_)/V(1-hexene) = 300:1.

**Table 4:** Carbon number distribution and group composition of Harbin FCC middle gasoline.

Carbon Number	Group Composition in Product (wt%)							Total (wt%)
	P	I	L-O	B-O	C-O	N	A	
C_4_	0.01	0.01	0.02	0	0	0	0	0.04
C_5_	0.10	0.15	0.22	0.58	0	0	0	1.05
C_6_	2.93	15.67	3.93	7.13	1.89	4.33	1.18	37.06
C_7_	1.88	15.31	1.57	5.92	0.50	8.84	6.74	40.76
C_8_	0	9.51	0.55	2.64	0	4.21	1.41	18.32
C_9_	0	1.29	0.45	0.89	0	0.02	0	2.65
C_10_	0	0	0	0.03	0	0	0.07	0.10
C_11_	0	0.02	0	0	0	0	0	0.02
Total	4.92	41.96	6.74	17.19	2.39	17.40	9.40	100

**Table 5:** Group composition and RON changes in hydro-upgrading products at different reaction temperatures *.

Reaction Temperature	Group Composition Changes in Product (wt%)							RON Changes
	P	I	L-O	B-O	C-O	N	A	
320 °C	2.48	1.96	−1.83	−5.20	−1.84	2.07	2.36	−0.42
340 °C	2.94	3.99	−4.17	−6.82	−2.04	2.15	3.94	−0.83
360 °C	3.10	5.64	−5.12	−9.99	−2.21	1.37	7.22	−0.29
380 °C	2.64	4.29	−5.93	−10.80	−2.32	0.73	11.40	2.60

* The other reaction conditions: P = 1.0 MPa; LHSV = 1.5 h^−1^; V(H_2_)/V(gasoline) = 300:1.

**Table 10:** Comparison between experimental and calculated data of FCC middle gasoline hydro-upgrading.

Component		Hydrocarbon Group Composition (wt%)						
		P	I	L-O	B-O	C-O	N	A
320 °C	y_ie_	7.40	43.92	4.92	11.99	0.55	19.47	11.76
	y_ic_	7.17	43.60	4.90	12.00	0.54	19.59	12.20
	RE (%)	−3.11	−0.73	−0.41	0.08	−1.82	0.62	3.74
340 °C	y_ie_	7.86	45.95	2.57	10.37	0.35	19.55	13.34
	y_ic_	7.91	45.80	2.54	10.48	0.36	19.56	13.35
	RE (%)	0.64	−0.33	−1.17	1.06	2.86	0.05	0.07
360 °C	y_ie_	8.02	47.60	1.62	7.20	0.18	18.77	16.62
	y_ic_	7.99	47.27	1.64	7.54	0.18	18.70	16.70
	RE (%)	−0.37	−0.69	1.23	4.72	0	−0.37	0.48
380 °C	y_ie_	7.56	46.25	0.81	6.39	0.07	18.13	20.80
	y_ic_	7.72	47.50	0.80	6.12	0.07	17.27	20.53
	RE (%)	2.12	2.70	−1.23	−4.23	0	−4.74	−1.30

**Table 3:** Carbon number distribution in the product with 1-hexene as feedstock at 1.5 h^−1^ and 3.0 h^−1^ *.

Carbon Number	Group Composition in Product (wt%) (LHSV = 1.5 h^−1^)							Group Composition in Product (wt%) (LHSV = 3.0 h^−1^)						
	P	I	L-O	B-O	C-O	N	A	P	I	L-O	B-O	C-O	N	A
≤C_3_	7.66	0	0.04	0	0	0	0	3.95	0	0.01	0	0	0	0
C_4_	7.90	11.24	0.41	0.62	0	0	0	2.23	4.12	3.11	4.74	0	0	0
C_5_	6.01	10.45	0.09	0.68	0	0.12	0	2.30	4.41	1.06	8.30	0.00	1.40	0
C_6_	3.80	10.15	0.12	0.27	0.19	1.25	0	3.57	7.27	0.99	5.32	0.51	0.99	0
C_7_	1.04	5.16	0	0.32	1.00	1.43	2.02	0.23	3.83	0.02	4.73	0.45	2.97	0.26
C_8_	0.35	2.90	0	0.37	1.78	1.37	3.44	1.12	2.52	0	1.20	5.52	2.21	0.50
C_9_	0.18	1.41	0	0.58	2.35	0.58	4.47	0.18	1.76	0	1.85	1.85	1.60	0.47
≥C_10_	0.11	1.68	0	1.79	0.48	0.53	3.66	0.33	3.16	0	1.99	0.98	2.83	3.16

* The other reaction conditions: T = 340 °C; P = 1.0 MPa; V(H_2_)/V(1-hexene) = 300:1.

**Table 6:** Changes in PINA (wt%) with carbon number of hydro-upgrading products at different reaction temperatures.

Carbon Number	320 °C				340 °C				360 °C				380 °C			
	P	I	N	A	P	I	N	A	P	I	N	A	P	I	N	A
C_4_	0.13	0.13	0	0	0.23	0.25	0	0	0.48	0.49	0	0	1.01	1.26	0	0
C_5_	0.28	0.36	0	0	0.45	0.62	0	0	0.81	1.13	0	0	1.37	1.89	0	0
C_6_	−0.13	−0.80	0.01	−0.96	0.02	0.01	0.21	−0.97	0.06	0.36	0.23	−0.98	−0.24	0.25	−0.13	−1.06
C_7_	0.18	0.79	0.93	0.12	0.19	1.27	0.59	−0.01	0.03	1.26	0.74	0.06	−0.53	0.38	0.26	0.61
C_8_	1.72	1.10	0.28	2.71	1.58	1.10	0.47	3.28	1.42	1.95	−0.42	4.97	1.04	0.26	−0.05	7.59
C_9_	0.19	−0.06	0.77	0.17	0.23	0.27	0.89	0.58	0.09	0.20	0.81	0.91	0	0.01	0.66	1.38
C_10_	0.12	0	0	0.33	0.25	0.10	0	0.67	0.21	0	0	1.77	0	0	0	2.42
C_11_	0	0.05	0	0	0	0.04	0	0	0	−0.02	0	0	0	−0.02	0	0.27
C_12_	0	0.39	0.08	0	0	0.34	0	0.39	0	0.28	0	0.50	0	0.12	0	0.19
C_13_	0	0	0	0	0	0	0	0	0	0	0	0	0	0.15	0	0

**Table 7:** Changes in olefins(wt%) with carbon number of hydro-upgrading products at different reaction temperatures.

Carbon Number	320 °C			340 °C			360 °C			380 °C		
	L-O	B-O	C-O	L-O	B-O	C-O	L-O	B-O	C-O	L-O	B-O	C-O
C_4_	0.88	0	0	0.82	0	0	0.63	0	0	0.41	0	0
C_5_	0.42	1.73	0	0.31	1.21	0	0.11	0.57	0	−0.03	−0.05	0
C_6_	−3.47	−5.46	−1.44	−3.64	−6.06	−1.62	−3.84	−6.55	−1.71	−3.89	−6.85	−1.82
C_7_	−0.86	−3.36	−0.40	−1.06	−3.71	−0.42	−1.27	−4.54	−0.50	−1.46	−4.63	−0.50
C_8_	1.47	1.31	0	−0.32	1.22	0	−0.47	0.63	0	−0.55	−0.32	0
C_9_	−0.27	0.05	0	−0.27	−0.28	0	−0.28	−0.46	0	−0.41	−0.73	0
C_10_	0	0.06	0	0	0.10	0	0	−0.03	0	0	1.25	0
C_11_	0	0.48	0	0	0.70	0	0	0.38	0	0	0.42	0

**Table 8:** Conversion behavior of C_6_ and C_7_ olefins at different reaction temperatures.

Carbon Number	Olefins Type	Olefins Name	Feed (wt%)	Product (wt%)			
				320 °C	340 °C	360 °C	380 °C
C_6_	L-O	1-hexene	0.37	0	0	0	0
		2-hexene,3-hexene	3.56	0.46	0.29	0.09	0.05
	B-O	2-methyl-1-pentene 4-methyl-1-pentene	1.71	0.32	0.21	0.13	0.06
		methyl-2-pentene 4-methyl-2-pentene methyl-3-pentene	5.28	1.35	0.86	0.46	0.22
		2,3-dimethyl-1-butene	0.15	0	0	0	0
	C-O	1-methylcyclopentene 3-methylcyclopentene	1.89	0.45	0.27	0.18	07
C_7_	L-O	2-heptene, 3-heptene	1.57	0.71	0.51	0.30	0.11
	B-O	3-methyl-1-hexene 4-methyl-1-hexene	0.24	0.07	0.06	0	0
		methyl-3-hexene 3-methyl-2-hexene	2.76	1.22	0.80	0.38	0.16
		2-ethyl-1-pentene 3-ethyl-1-pentene	1.55	1.18	0.99	0.93	1.07
		ethyl-2-pentene 4,4-dimethyl-2-pentene	0.41	0.10	0.06	0	0
		2.3.3-trimethylbutene 2-ethyl-3-methyl-1-butene	0.11	0	0.06	0.07	0.07
		Others	0.85	0	0.14	0	0
	C-O	3-ethylcyclopentene 1-ethylcyclopentene	0.25	0.10	0.08	0	0
		1-methylcyclohexene	0.24	0	0	0	0

**Table 9:** Calculation results of parameters of a kinetic model for FCC middle gasoline hydro-upgrading.

No.	Reaction Path	Rate Constant k (cm^3^·g^−1^·s ^−1^)				Ea (kJ·mol^−1^)	A (cm^3^·g^−1^·h^−1^)
		320 °C	340 °C	360 °C	380 °C		
1	P → I	0.30	2.54	4.77	6.41	160.17	6.08 × 10^13^
2	N → I	0.27	1.51	3.15	4.47	148.22	4.28 × 10^12^
3	N → P	3.76	4.98	5.79	6.12	33.65	3.52 × 10^3^
4	N → A	0.46	2.24	9.64	28.98	224.03	2.61 × 10^19^
5	A → N	16.22	21.09	24.80	34.06	38.31	3.80 × 10^4^
6	B-O → I	8.45	19.55	30.07	32.84	73.14	2.76 × 10^7^
7	L-O → P	18.59	38.31	50.50	63.50	64.18	9.47 × 10^6^
8	C-O → N	60.40	80.28	113.70	163.40	53.48	3.00 × 10^6^
9	C-O → A	58.39	75.45	109.32	160.72	54.63	3.61 × 10^6^
10	L-O → B-O	0.50	16.45	26.21	48.90	90.35	7.94 × 10^8^
11	B-O → C-O	0.14	0.20	0.23	0.39	53.08	6.37 × 10^3^
12	L-O → C-O	0.11	0.15	0.24	0.46	74.94	4.06 × 10^5^
13	L-O → A	5.72	24.04	41.00	69.99	88.79	8.77 × 10^8^
14	B-O → A	19.64	25.25	46.06	67.01	68.70	2.05 × 10^7^

**Table 11:** Properties of La-Ni-Zn/H-ZSM-5 hydro-upgrading catalyst.

Item	Value	Unit
packing density	0.60	g/mL
micropore surface area ^a^	225	m^2^/g
external surface area ^b^	48	m^2^/g
micropore volume ^c^	0.10	mL/g
mesopore volume ^d^	0.15	mL/g
average pore diameter ^e^	3.63	nm
weak Lewis acidity	94.6	µmol/g
weak Brönsted acidity	22.7	µmol/g
medium and strong Lewis acidity	80.7	µmol/g
medium and strong Brönsted acidity	9.5	µmol/g
Metal oxide content ^f^	1.1%La_2_O_3_/4.8%ZnO/5.5%NiO	wt%

^a^ Micropore surface area calculated by the BET method. ^b^ External surface area calculated by the t-plot method. ^c^ Micropore volume calculated by t-plot method. ^d^ Mesopore volume calculated by subtracting the micropore volume from the total volume. ^e^ The average pore diameter was the adsorption average pore width (4 V/A by BET). ^f^ The compositions of samples were tested by X-ray Fluorescence Spectrometer.

**Table 12:** Carbon number distribution and group composition of Harbin FCC gasoline.

Carbon Number	Group Composition in Product (wt%)							Total (wt%)
	P	I	L-O	B-O	C-O	N	A	
C_4_	0.31	0.08	1.26	0	0	0	0	1.65
C_5_	1.61	9.96	3.65	6.52	0.04	0	0	21.78
C_6_	1.37	10.83	1.89	3.77	0.93	2.07	0.56	21.42
C_7_	0.79	6.16	0.89	2.54	0.07	3.49	2.84	16.78
C_8_	0.78	3.98	0.38	1.42	0	1.69	6.53	14.78
C_9_	0.34	2.26	0.10	0.48	0	1.35	7.72	12.25
C_10_	0.32	1.19	0	0.27	0	0.06	4.79	6.63
C_11_	0.24	1.51	0.19	0.35	0	0.04	0.75	3.08
C_12_	0.10	0.92	0	0	0	0	0.61	1.63
Total	5.86	36.89	8.36	15.35	1.04	8.70	23.80	100

## Data Availability

Data are contained within the article.
